# Cell surface expression of homomeric GABA_A_ receptors depends on single residues in subunit transmembrane domains

**DOI:** 10.1074/jbc.RA118.002792

**Published:** 2018-07-09

**Authors:** Saad Hannan, Trevor G. Smart

**Affiliations:** From the Department of Neuroscience, Physiology, and Pharmacology, University College London, Gower Street, London WC1E 6BT, United Kingdom

**Keywords:** GABA receptor, trafficking, homology modeling, receptor, ion channel, homomers, inhibition, receptor assembly

## Abstract

Cell surface expression of type A GABA receptors (GABA_A_Rs) is a critical determinant of the efficacy of inhibitory neurotransmission. Pentameric GABA_A_Rs are assembled from a large pool of subunits according to precise co-assembly rules that limit the extent of receptor structural diversity. These rules ensure that particular subunits, such as ρ1 and β3, form functional cell surface ion channels when expressed alone in heterologous systems, whereas other brain-abundant subunits, such as α and γ, are retained within intracellular compartments. Why some of the most abundant GABA_A_R subunits fail to form homomeric ion channels is unknown. Normally, surface expression of α and γ subunits requires co-assembly with β subunits via interactions between their N-terminal sequences in the endoplasmic reticulum. Here, using molecular biology, imaging, and electrophysiology with GABA_A_R chimeras, we have identified two critical residues in the transmembrane domains of α and γ subunits, which, when substituted for their ρ1 counterparts, permit cell surface expression as homomers. Consistent with this, substitution of the ρ1 transmembrane residues for the α subunit equivalents reduced surface expression and altered channel gating, highlighting their importance for GABA_A_R trafficking and signaling. Although not ligand-gated, the formation of α and γ homomeric ion channels at the cell surface was revealed by incorporating a mutation that imparts the functional signature of spontaneous channel activity. Our study identifies two single transmembrane residues that enable homomeric GABA_A_R subunit cell surface trafficking and demonstrates that α and γ subunits can form functional ion channels.

## Introduction

GABA plays a central role in neurotransmission by exerting inhibitory control over neuronal excitation. Dysfunctional GABAergic neurotransmission is associated with many neurological conditions, including epilepsy (1), anxiety (2), and neurodevelopmental disorders (3). The cellular effects of GABA are orchestrated by ionotropic type A GABA receptors (GABA_A_Rs)^3^ and by metabotropic GABA_B_ receptors. The activation of inhibitory Cl^−^ and K^+^ conductances via these two receptor classes hyperpolarize and electrically shunt neurons to control excitation (4). GABA_A_Rs are expressed within and outside of inhibitory synapses, and their cell surface expression levels critically determine inhibitory efficacy.

GABA_A_Rs are pentamers constructed from combinations of 19 subunits (α1–6, β1–3, γ1–3, δ, ρ1–3, ϵ, π, and θ) (5, 6). Their abundance depends on the brain region and cellular location. The prototypical GABA_A_R is a heteropentamer comprising 2α and 2β with either γ or δ subunits (7). In heterologous expression systems (HEK293 cells or *Xenopus* oocytes), α and the long isoform of γ subunits (γ2L) do not travel to the cell surface alone, as determined by biochemical, imaging, and electrophysiological methods (8–10). Formation of functional cell surface ion channels requires co-assembly with β subunits in the endoplasmic reticulum (8, 9). Assembly of the pentamer is proposed to stabilize receptor conformation, permitting its trafficking to the cell surface (11). To date, only ρ1 and β3 subunits have been reported to be robustly expressed at the cell surface of heterologous expression systems as functional homomers (12–16). In addition, ϵ subunits can also traffic to the cell surface but do not form functional ion channels (17). The trafficking itineraries of ρ1 and β3 functional homomers indicate that, normally, α and γ subunits are internally sequestered in the absence of β subunits, suggesting the presence of intracellular retention signals.

Several sequence motifs have been described for α, β, and γ subunits that facilitate heteropentameric assembly (18–21). These motifs are located in the N-terminal extracellular domains (ECDs), and their deletion or substitution abolishes oligomerization in biochemical assays (18–20, 22–24). The location of these motifs implies that interfacial subunit interactions are important for receptor trafficking to the cell surface. However, what prevents surface expression of the most abundant GABA_A_R α and γ subunits in the absence of heteromeric subunit co-assembly remains unknown.

We addressed this issue by identifying residues that affected homomeric GABA_A_R surface trafficking using imaging with a fluorophore-linked α-bungarotoxin (BgTx) bound to a mimotope representing the BgTx-binding site (BBS) (25–27) inserted into the subunit's ECD. The role of the transmembrane domain (TMD) was examined in assembly using chimeras, and the intracellular domains (ICDs) between M1–M2 and M3–M4 were assessed using domain swaps with the prokaryotic receptor homologue from *Gloeobacter violaceus*, GLIC. Our results provide a new framework for understanding homomeric GABA_A_R trafficking based on single TMD residues that enable functional cell surface assembly of α and γ subunit homomers.

## Results

### Switching domains in α1 subunits

**Figure 1.**
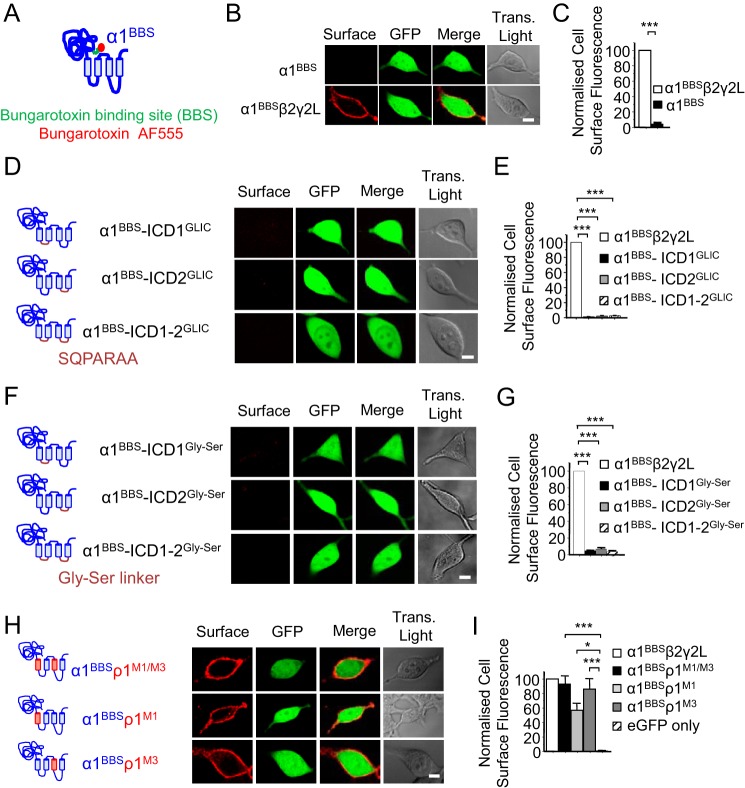
**Transmembrane domains 1 and 3 control the surface expression of homomeric GABA_A_R α1 subunits.** *A*, schematic of the mouse GABA_A_R α1 subunit, showing the BBS (*green*) and bound α-BgTx coupled to AF555 (*red*). *B*, confocal images for cell surface α1^BBS^ expressed alone or with β2 and γ2L subunits in HEK293 cells. *C*, analysis of cell surface expression for α1^BBS^ and α1^BBS^β2γ2L in HEK293 cells. Note that α1 subunits reach the surface only with β2 and γ2 subunits. *D*, schematic of receptor constructs and confocal images for α1 subunits with ICD1, ICD2, or ICD1–2 replaced by the GLIC M3–M4 sequence -SQPARAA- (*purple*). *E*, cell surface expression of α1^BBS^β2γ2L (from *B*) and α1 subunit ICD1, ICD2, and ICD1–2 substituted with GLIC M3–M4. *F*, schematic of receptor constructs and confocal images for α1 subunits with ICD1, ICD2, or ICD1–2 replaced by a multiple Gly-Ser flexible linker, -GGSSGGSS- (*purple*). *G*, cell surface expression of α1^BBS^β2γ2L and the α1 subunit with ICD1, ICD2, and ICD1–2 substituted by a Gly-Ser linker. *H*, confocal images for α1^BBS^–ρ1 chimeras, including α1^BBS^–ρ1^M1/M3^, α1^BBS^–ρ1^M1^, and α1^BBS^–ρ1^M3^. *I*, cell surface fluorescence for α1^BBS^–ρ1 chimeras. These and similar data in other figures are normalized to cell surface labeling for WT α1^BBS^β2γ2L receptors. Data in all bar charts represent mean ± S.E.. *, *p* < 0.05; ***, *p* < 0.001; *n* = 6–12; two-tailed unpaired *t* test (*C*) and one-way ANOVA (*E* and *G*). *Scale bars* = 5 μm

To understand why the cell surface expression profiles for α and ρ subunits differ, we created chimeras incorporating domain switches. We used the prototypic α1 subunit incorporating a BBS (α1^BBS^; Fig. 1*A*) to allow specific labeling with Alexa 555–conjugated BgTx (Fig. S1, *A* and *B*). As expected (20, 28), α1^BBS^ did not express on the surface of HEK293 cells, evident by an absence of BgTx-AF555 labeling at the cell periphery; however, intracellular fluorescence was observed in permeabilized cells (Fig. S1, *A* and *B*). By contrast, co-expression of α1 with β and γ subunits permitted robust surface expression (*p* < 0.001; Fig. 1, *B* and *C*). The inserted BBS motif did not unduly affect receptor function because activation by GABA of αβγ receptors in HEK293 cells revealed only a small (2.6-fold) decrease in GABA sensitivity compared with WT receptors (EC_50_ α1β2γ2L 8.3 ± 1.4 μm, *n* = 6; α1^BBS^β2γ2L 20.4 ± 3.1 μm, *n* = 12; Fig. S1*C*).

The importance of the α1^BBS^ ICDs (between M1–M2 (ICD1) and M3–M4 (ICD2)) for surface expression was investigated next by substitution with the ICD sequence from GLIC, -SQPARAA-. GLIC is a part of the pentameric ligand-gated ion channel family (29). The GLIC heptapeptide ICD is frequently used in structural studies to stabilize GABA_A_Rs for X-ray crystallization (30–31) and is therefore also useful to explore the properties of the α subunit ICDs. In addition, we studied the role of ICDs in assembly using an alternative set of constructs where the ICDs were replaced with Gly-Ser flexible linkers. The critical nature of this region is further demonstrated by the substitution of intracellular residues at the base of the transmembrane α helices, which can affect signaling via GABA_A_Rs (32).

Both GLIC ICD- and Gly-Ser–containing α1 subunits were absent from the cell surface, with low fluorescence intensities similar to background, and significantly lower compared with α1^BBS^β2γ2L receptors (*p* < 0.001; Fig. 1, *D–G*) even though intracellular labeling of permeabilized cells was observed (Fig. S2, *A* and *B*) and the GLIC and Gly-Ser ICD–containing chimeras expressed on the cell surface with β2γ2L subunits (Fig. S2, *C* and *D*). We initially considered that the ICD may be important for trafficking because γ2L subunits are retained in the ER, contrasting with the alternatively spliced γ2S isoform that differs only by the deletion of an eight-amino acid motif in the ICD (10).

Nevertheless, substitution of the ICDs separately or together, with either GLIC or Gly-Ser linkers, did not permit α subunit access to the cell surface. We also considered whether the GLIC heptapeptide induced a dominant negative effect on expression, but this is unlikely given the successful expression of β3 homomers incorporating the GLIC ICD (30) and the clear cell surface expression of α and γ2L subunits, also incorporating GLIC ICDs, as αβγ heteromers. We therefore eliminated the ICDs from further investigation as a reason for intracellular retention of α and γ2L subunits. We took this decision even though we know from previous work that γ2S can traffic to the cell surface but then fails to form oligomers or functional ion channels, and it is significantly internalized (10).

Given that ρ1 subunits form cell surface homomers, the role of the TMD in subunit trafficking was assessed by forming domain swap chimeras between the α1^BBS^ and ρ1 subunits. These include α1^BBS^-ρ1^M1–4^; α1^BBS^ and ρ1 M1 and M2 (α1^BBS^-ρ1^M1/2^), α1^BBS^ and ρ1 ICD2 + M4 (α1^BBS^-ρ1^ICD2-M4^), and α1^BBS^ ECD and ρ1 M4 (α1^BBS^-ρ1^M4^). However, all of these constructs failed to traffic to the cell surface, as determined by using BgTx–Alexa 555 (Fig. S3, *A* and *B*, *p* < 0.001). Nevertheless, intracellular labeling was evident in permeabilized cells, suggesting that these chimeras were retained in intracellular compartments (Fig. S3, *C* and *D*, *p* < 0.001). By contrast, α1-ρ1 chimeras composed of α1^BBS^ with ρ1 M3, ICD2, and M4 (α1^BBS^-ρ1^M3-M4^) and α1^BBS^ with ρ1 M1 and M3 (α1^BBS^-ρ1^M1/M3^) both showed clear surface expression (Fig. S3, *A* and *B* and Fig. 1, *H* and *I*; *p* < 0.001). Given that α1^BBS^-ρ1^M4^ and α1^BBS^-ρ1^M1/M2^ were not assembled at the cell surface, we next examined a chimera formed from α1^BBS^ and just ρ1 M3 (α1^BBS^-ρ1^M3^). This also showed robust surface expression (Fig. 1, *H* and *I*; *p* < 0.001) suggesting an important role for M3 residues in surface trafficking of α1 subunits.

In addition, because α1^BBS^-ρ1^M1/M3^ was expressed at the cell surface, we examined the contribution of M1 to surface trafficking. A new chimera comprising α1^BBS^ ECD and ρ1 M1 (α1^BBS^-ρ1^M1^) also revealed strong cell surface labeling (Fig. 1, *H* and *I*; *p* < 0.05). Together, these results reveal the existence of two discrete areas in M1 and M3 of the α1 TMD that control cell surface expression.

### Two TMD residues independently control cell surface expression of α1 subunits

**Figure 2.**
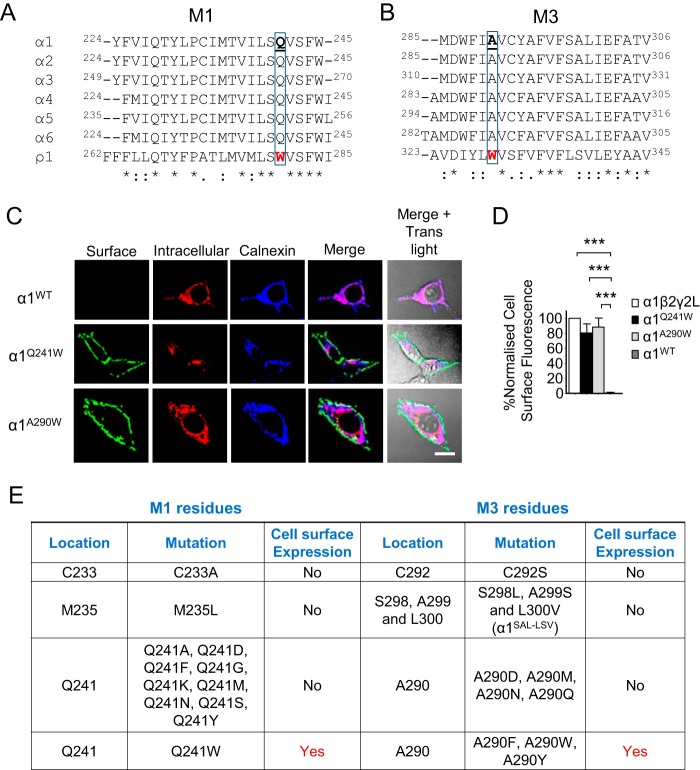
**Two critical amino acids control the cell surface expression of homomeric GABA_A_R α1 subunits.** *A*, primary sequence alignment of mouse α1–6 and ρ1 subunit M1 domains. The highly conserved Gln in α^M1^ replaced by Trp (*red*) in ρ1^M1^ is *boxed. B*, similar alignment for M3 reveals a highly conserved Ala residue in α^M3^ replaced by Trp in ρ1^M3^. *Numbers* refer to mature proteins. *C*, confocal images of cell surface labeling for α1^Q241W^ and α1^A290W^ compared with ER-retained WT α1 in HEK293 cells. *D*, cell surface fluorescence for α1^WT^, α1^Q241W^ and α1^A290W^ homomers, and α1β2γ2L. Intracellular receptors in the ER were identified by membrane permeabilization and calnexin co-labeling. *E*, summary of M1 (*left*) and M3 (*right*) mutations used to identify motifs that affect the trafficking of α1 homomers. Q241W in M1 or A290F/A290Y/A290W in M3 resulted in cell surface expression of α1 subunits. ***, *p* < 0.001; *n* = 8–14; two-tailed unpaired *t* test (*C*) and one-way ANOVA (*E* and *G*). *Scale bars* = 5 μm.

To identify the critical amino acids involved in cell surface expression of the α1^BBS^-ρ1^M1/M3^ chimeras, we compared the primary sequences for M1 and M3 between α1–6 and ρ1 subunits. Although the M1 sequences were highly conserved, nonhomologous exchanges were identified (Fig. 2*A*). Using the mouse α1 subunit as a template, we sequentially substituted three highly conserved M1 residues with nonhomologous equivalents from ρ1^M1^. Two of these substitutions, C233A and M235L, did not enable α1 subunits to reach the cell surface (Fig. S4, *A* and *B*), even though intracellular labeling was observed (Fig. S4, *C* and *D*). However, Q241W, which is located at the base of M1, caused robust cell surface expression (Fig. 2, *C–E*; Fig. S4, *A* and *B*; *p* < 0.001), indicating a key role in the surface trafficking of α1 subunits.

Comparing sequences for M3 across α1–6 and ρ1 subunits revealed similar high levels of residue conservation (Fig. 2*B*). Substituting several nonhomologous residues in α1^M3^ for their ρ1^M3^ equivalents, including C292S and the triple switch S298L, A299S, and L300V (α1^SAL-LSV^), revealed no effect on cell surface expression, only intracellular retention (Fig. 2, *C* and *D*, and Fig. S4; *p* < 0.001). However, the substitution α1^A290W^, which is located at the top of M3, enabled robust surface expression comparable with that for α1-containing heteromers (Fig. 2, *C* and *D*, and Fig. S4, *A* and *B*; *p* < 0.001). Thus, position 290 in M3 is another critical determinant for surface expression of α1 subunits.

To unequivocally demonstrate that these point mutations permit cell surface expression of α1 subunits, we used double labeling with different fluorophores coupled to BgTx. HEK293 cells expressing BBS-tagged WT α1 (α1^WT^), α1^Q241W^, or α1^A290W^ were incubated in Alexa Fluor 488–BgTx. Then, after fixation and permeabilization, we incubated with Alexa Fluor 555–BgTx and an antibody for the endoplasmic reticulum (ER) marker calnexin. This revealed that, although α1^Q241W^ and α1^A290W^ reach the cell surface, WT α1 subunits are retained in the ER (Fig. 2, *C* and *D*; *p* < 0.001).

To determine the nature of the residues enabling cell surface expression of α1 subunits, Gln-241 was exchanged for alternatives with small (Gly), hydrophobic (Ala, Met), positively charged (Lys), negatively charged (Asp), polar (Asn, Ser), or aromatic (Phe, Tyr) side chains. None of these substitutions permitted cell surface expression compared with Q241W (*p* < 0.001), although intracellular labeling was evident for all mutants (Fig. 2*E* and Fig. S5; *p* > 0.05). Similar examination of Ala-290 by substitution with Asp, Met, Gln, or Asn also failed to promote cell surface expression of α1 subunits (*p* < 0.001), although again, intracellular labeling was evident, suggesting that these subunits fold correctly in the ER (Fig. 2*E* and Fig. S6; *p* > 0.05). Interestingly, substitution of Ala-290 by Phe or Tyr resulted in cell surface expression, albeit at a lower level compared with A290W (Fig. S6, *A* and *B*). Together, these results indicate that substitution of the highly conserved M1 residue to tryptophan (from ρ1) or the M3 residue to aromatic amino acids (tryptophan in ρ1), allows cell surface expression of α1 subunits.

### α1 subunits form functional homomeric ion channels

**Figure 3.**
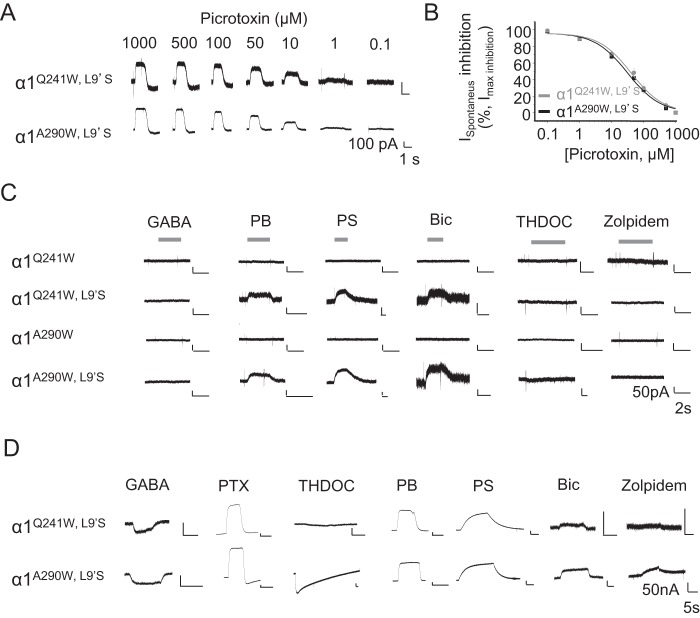
**GABA_A_R α subunits form functional cell surface ion channels.** *A* and *B*, spontaneous membrane currents revealed by picrotoxin (PTX) (*A*) and concentration inhibition curves for PTX in HEK293 cells expressing α1^Q241W, L9'S^ (IC_50_, 40 ± 8 μm; *n* = 8) and α1^A290W, L9'S^ (IC_50_, 31 ± 5 μm; *n* = 15) (*B*). *C*, membrane currents recorded in HEK293 cells for α1 homomers with or without the L9'S substitution after applying: GABA (1 mm), pentobarbitone (PB, 50 μm), pregnenolone sulfate (PS, 10 μm), bicuculline (Bic, 200 μm), THDOC (10 μm), or zolpidem (100 μm). *D*, membrane currents for α1^L9'S^ receptors recorded in *Xenopus* oocytes in response to GABA (100 mm), PTX (1 mm), THDOC (10 μm), PB (1 mm), PS (10 μm), Bic (200 μm), or zolpidem (100 μm).

To determine whether cell surface α1^Q241W^ and α1^A290W^ formed functional ion channels, a leucine residue in M2 (9') was exchanged for serine (L9'S). This was necessary to confer spontaneous channel activation (33) because α homomers lack a GABA binding site because of the absence of β subunits. Cells expressing either α1^Q241W,L9'S^ or α1^A290W,L9'S^ exhibited spontaneous activity, as revealed by the GABA_A_R channel blocker picrotoxin, which caused inhibition in a concentration-dependent manner (Fig. 3, *A* and *B*).

When expressed in HEK293 cells, α1^Q241W^ or α1^A290W^ homomers were not activated by GABA (1 mm), pentobarbitone (50 μm), the neurosteroid tetrahydrodeoxycorticosterone (THDOC, 10 μm), or zolpidem (100 μm), which has been reported to bind to α–α interfaces (34). However, the spontaneous current induced by L9′S was inhibited by pentobarbitone (50 μm), pregnenolone sulfate (10 μm), and bicuculline (200 μm) but not by GABA, THDOC, or zolpidem (Fig. 3*C*). When homomeric α1 receptors lacking L9'S were expressed in *Xenopus* oocytes, no activation of α1^Q241W^ and α1^A290W^ was observed with GABA (100 mm), pentobarbitone (1 mm), or zolpidem (100 μm) (data not shown). However, by including L9'S, GABA evoked small currents for α1^Q241W,L9'S^ and α1^A290W,L9'S^ homomers, and pentobarbitone, pregnenolone sulfate, bicuculline, and zolpidem all blocked the spontaneous currents (Fig. 3*D*). Interestingly, THDOC (10 μm) caused a large activation of α1^A290W,L9'S^ but not α1^Q241W,L9'S^, followed by slow deactivation (35). Glutamine 241 forms a key part of the neurosteroid binding site (35), and its substitution with tryptophan (α1^Q241W^) can mimic a neurosteroid-bound state. This may enable the release of α1 subunits from intracellular compartments to the cell surface. However, treating HEK293 cells expressing just α1 subunits with THDOC (up to 10 μm) did not enable α1 subunit trafficking to the cell surface (Fig. S7). These results indicate that α1 subunits incorporating single mutations in the TMD can assemble into functional homomeric ion channels on the cell membrane.

### A conserved amino acid controls the cell surface expression of γ2

**Figure 4.**
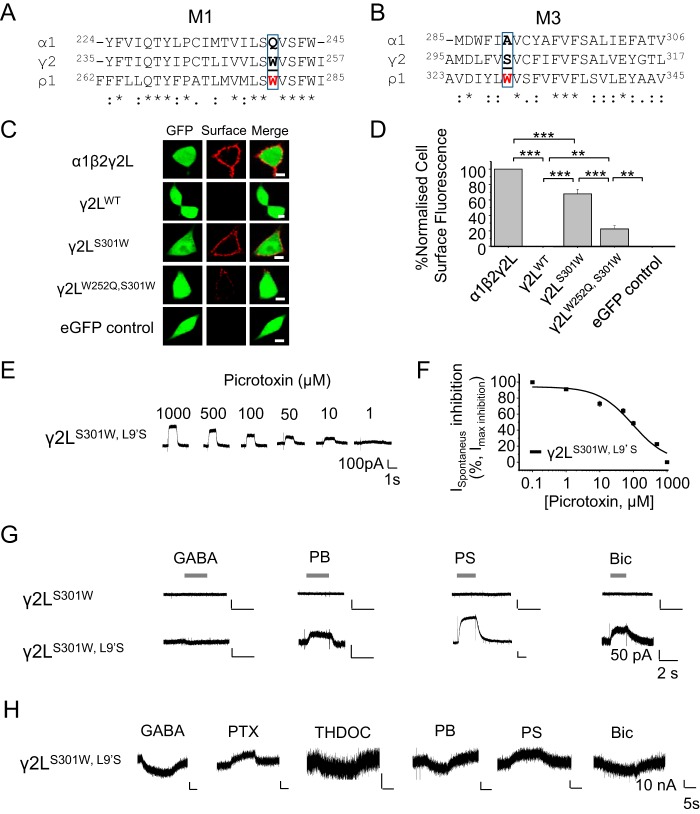
**GABA_A_R γ2 subunits form functional cell surface ion channels.** *A*, primary sequence alignments for γ2 with α1 and ρ1 subunits identifying a tryptophan (*boxed*, *bold underlined*) in M1 also found in ρ1^M1^. *B*, a *s*imilar alignment identifies a serine in γ2^M3^ that corresponds to Trp in ρ1^M3^. *C*, confocal images for γ2L^S301W^ reveal cell surface expression compared with γ2L^WT^, γ2L^W252Q,S301W^, and the eGFP control in HEK293 cells. *D*, cell surface fluorescence for γ2^WT^, γ2^S301W^ and γ2L^W252Q,S301W^, α1β2γ2L, and eGFP. *E*, inhibition of spontaneous currents by PTX in HEK293 cells expressing γ2L^S301W, L9'S^. *F*, PTX concentration inhibition curves for γ2L^S301W, L9'S^ (IC_50_, 96 ± 32 μm; *n* = 6). *G*, membrane currents evoked in HEK293 cells expressing γ2L homomeric channels with or without the L9'S substitution in response to GABA (1 mm), pentobarbitone (50 μm), pregnenolone sulfate (10 μm), or bicuculline (200 μm). *H*, membrane currents for γ2L^S301W,^ ^L9'S^ in *Xenopus* oocytes after GABA (100 mm), PTX (1 mm), THDOC (10 μm), PB (1 mm), PS (10 μm), Bic (200 μm), or zolpidem (100 μm). **, *p* < 0.01, ***, *p* < 0.001, *n* = 5–15, one-way ANOVA. *Scale bar* = 5 μm.

The γ2L subunit, similar to α1, is also unable to access the plasma membrane as a functional homomer (9, 10, 36). Comparing the primary sequences in M1 and M3 across γ2L, α1, and ρ1 subunits revealed a tryptophan (Trp-252) in γ2^M1^ (present also in ρ1^M1^) in the same position as Gln-241 in α1^M1^ (Fig. 4*A*). In addition, in γ2^M3^, a serine (Ser-301) occupies the equivalent α1^A290^ site (Fig. 4*B*). Mutating Ser-301 to tryptophan (γ2L^S301W^) allowed γ2L subunits to access the cell surface, as evident from surface fluorescence after immunolabeling with a γ2 antibody. Similar surface labeling was evident for WT heteromeric αβγ receptors but not for WT γ2L alone (Fig. 4, *C* and *D*; *p* < 0.001). In addition, mutating the γ2^M1^ Trp-252 to glutamine in the γ2L^S301W^ background (γ2L^W252Q,S301W^) largely reduced but did not abolish surface expression of γ2L (Fig. 4, *C* and *D*; *p* < 0.01).

These results suggest that exchanging S301W in γ2L^M3^ enables access to the cell surface and that mutating another γ2L tryptophan in M1 (to Gln, the α1^Gln-241^-equivalent site) partly prevented this. This implies the existence of multiple trafficking determinants in addition to the ER retention motif present in the ICD of γ2L (10).

To determine whether γ2L subunits (like α1) can form functional ion channels, we used the L9'S switch in γ2L^S301W^ and expressed these receptors (γ2L^S301W,L9'S^) in HEK293 cells. Spontaneous channel activity was evident and blocked by picrotoxin (Fig. 4, *E* and *F*), confirming that γ2L homomers formed functional channels. In addition, γ2L^S301W^ homomers were not activated by GABA or pentobarbitone, but spontaneous activity was inhibited by pentobarbitone, pregnenolone sulfate, and bicuculline but not by GABA (Fig. 4*G*). By comparison, expression of γ2L^S301W,L9'S^ in oocytes produced spontaneous currents inhibited by picrotoxin and pregnenolone sulfate. In addition, GABA, THDOC, pentobarbitone, and bicuculline caused minimal activation of γ2L^S301W,L9'S^ (Fig. 4*H*). These results highlight the importance of single M3 residues for controlling the cell surface expression of γ2L subunits.

### Conserved TMD residues affect the cell surface expression and signaling of ρ1 homomers

**Figure 5.**
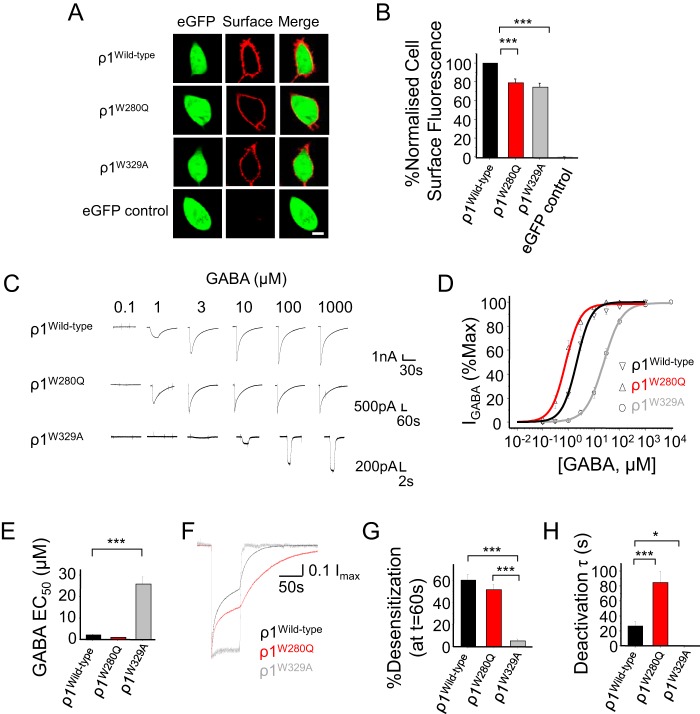
**Mutating M1 and M3 residues impairs trafficking and signaling of ρ1 homomers.** *A*, confocal images of cell surface expression for immunolabeled WT and mutant ρ1 receptors expressed in HEK293 cells. *B*, cell surface fluorescence for WT and mutant ρ1 receptors and eGFP controls. Data are normalized to labeling for WT ρ1 receptors. *C*, representative GABA-activated current profiles for WT and mutant ρ1 receptors expressed in HEK293 cells. *D*, concentration response profiles for WT and mutant ρ1 receptors. *E*, GABA EC_50_ values for activating WT and mutant ρ1 receptors. *F*, averaged activation, desensitization, and apparent deactivation waveforms for WT and mutant ρ1 receptors in response to 1 mm GABA (*n* = 5–8 currents). Waveforms were normalized to the peak current. *G*, extent of peak current desensitization at the end of GABA application (percent peak). *H*, weighted apparent deactivation time constants for WT and mutant ρ1 receptors. Deactivation data were normalized to the point at which GABA application ceased and fitted with an exponential function. *, *p* < 0.05; ***, *p* < 0.001; *n* = 5–10; one-way ANOVA. *Scale bars* = 5 μm.

Site-directed mutation of α1 and γ2L indicated the importance of two tryptophans in surface trafficking and formation of functional homomers. To investigate whether these TMD residues are important for surface expression of naturally occurring GABA_A_R homomers, we switched the ρ1 M1 and M3 tryptophans to their α subunit equivalents in ρ1^W280Q^ and ρ1^W329A^. Both mutations reduced but did not abolish ρ1 surface expression (Fig. 5, *A* and *B*; *p* < 0.001), suggesting that they play a lesser role in trafficking for ρ1 subunits.

We also examined the role of these TMD residues in GABA activation of ρ1 homomers. Differential effects were evident, with ρ1^W280Q^ increasing GABA potency by ∼2.5-fold (EC_50_ = 0.92 ± 0.17 μm, *n* = 5, *p* > 0.05; Fig. 5, *C–E*), whereas ρ1^W329A^ caused a 10-fold reduction (25.76 ± 3.39 μm, *n* = 10, *p* < 0.001), as observed previously (37), compared with WT ρ1 receptors (2.19 ± 0.65 μm, *n* = 7).

In addition, these ρ1 mutants exhibited changed desensitization and deactivation kinetics. Strikingly, mutating the M3 tryptophan (ρ1^W329A^) markedly reduced the extent of desensitization by 1 mm GABA (*n* = 5; Fig. 5, *F* and *G*) compared with WT ρ1 (*n* = 10, *p* < 0.001) and ρ1^W280Q^ (*n* = 13, *p* < 0.001). The ρ1^W329A^ substitution also caused a profound increase in the speed of current deactivation (τ = 0.86 ± 0.1 s, *n* = 7, *p* < 0.05; Fig. 5, *F* and *H*) compared with WT ρ1 (τ = 26.9 ± 5.6 s, *n* = 8) and ρ1^W280Q^, where deactivation was even slower (τ = 84.4 ± 15.1 s, *n* = 5, *p* < 0.001). These results indicate that the residues controlling homomeric receptor expression in α and γL subunits also have important trafficking and gating roles for ρ1 homomers depending on the substituted residue.

### Conserved TMD residues affect signaling of αβγ heteromers

**Figure 6.**
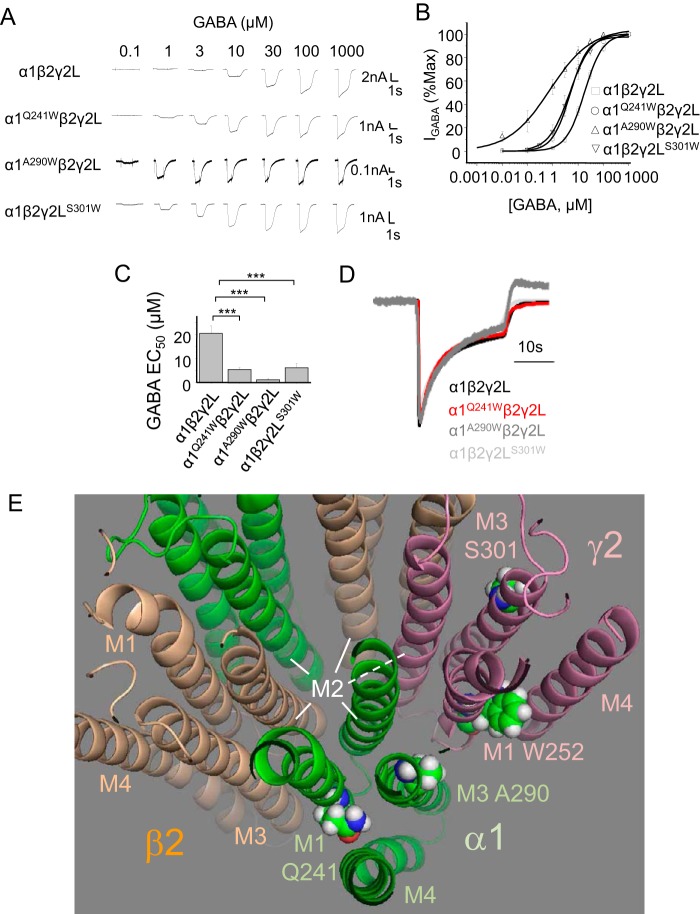
**Mutating M1 and M3 residues impairs GABA signaling via αβγ heteromers.** *A–C*, representative GABA-activated currents, concentration response curves, and EC_50_ values for WT and mutant αβγ receptors. ***, *p* < 0.001; *n* = 7–12; one-way ANOVA. *D*, mean activation, desensitization, and apparent deactivation waveforms for WT and mutant αβγ receptors in response to 1 mm GABA. Waveforms are normalized to the peak current (*n* = 13–16 traces). *E*, tilted plan view of the TMD for a pentameric αβγ GABA_A_R homology model based on the β3 subunit crystal structure (PDB code 4COF) (30). A ring of M2 α helices from each subunit form the ion channel pore. The TMDs for α1 (*green*), β2 (*wheat*), and γ2 (*pink*) subunits are shown. Note the interfacial locations for M1 α1^Q241^ (interface β2–α1), M3 α1^A290^ (α1–γ2), M1 γ2^W252^ (α1–γ2), and M3 γ2^S301^ (γ2–β2).

Following the impact of the tryptophan substitutions on ρ1 receptor kinetics and GABA sensitivity, we next assessed their importance for GABA_A_R heteromers. GABA concentration response curves for αβγ receptors in HEK293 cells revealed increased GABA potency for α1^Q241W^β2γ2L (EC_50_ = 5.4 ± 0.8 μm, *n* = 12), α1^A290W^β2γ2L (1.2 ± 0.4 μm, *n* = 9), and α1β2γ2L^S301W^ (6.2 ± 1.8 μm, *n* = 7) (for all, *p* < 0.001) compared with WT α1β2γ2L (20.4 ± 3.1 μm, *n* = 12; Fig. 6, *A–C*).

The reduced EC_50_ values were not a consequence of αβ heteromers reflecting nonassembly of γ subunits (Fig. S8) because the mutant receptors lacked sensitivity to the αβ subtype–selective blocker Zn^2+^ at 10 μm (38, 39). Thus, the tryptophans are important for signaling via heteromeric GABA_A_Rs, although desensitization and deactivation appeared to be unchanged (Fig. 6*D*).

To determine the location of these critical TMD residues in GABA_A_Rs, we created a homology model based on the GABA β3 subunit crystal structure (30). Interestingly, the model reveals that the key TMD residues involved in α1 and γ2 subunit cell surface expression are located at interface sites between α–γ and β–α subunits (for α subunits) and at γ–β (for γ subunits) (Fig. 6*E*).

**Figure 7.**
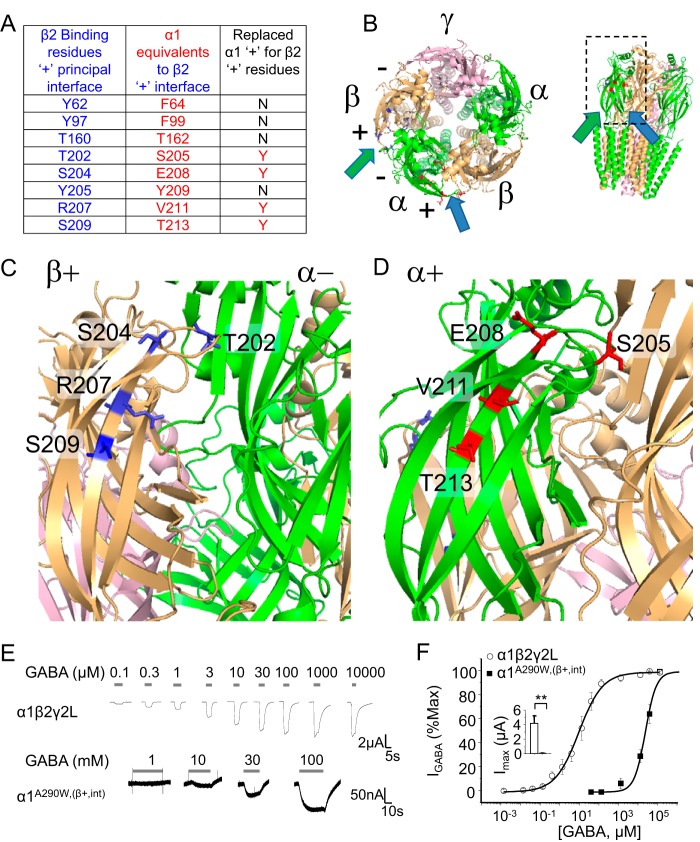
**Recreating the β–α subunit interface restores GABA sensitivity to α homomers.** *A*, panel of residues in β2 at the principal interface (+) that are involved in GABA binding in αβγ receptors. Corresponding residues at the α^+^ interface are shown. Note that 50% of these residues were exchanged in the α1 homomers as indicated (*N*, not exchanged; *Y*, yes, exchanged). *B*, plan and side views of a GABA_A_R homology model based on the β3 subunit crystal structure (PDB code 4COF) (30). The latter views are tilted to reveal the crucial binding residues. The *arrows* indicate the viewing directions shown in *C* and *D. C* shows the β^+^–α^−^ interface (*green arrows* in *B*) and the four residues (*blue*) from the β2 subunit that were incorporated into the α1 subunit. *D*, a similar view of a subunit interface but now showing the α+ side (*blue arrows* in *B*) and the selected four residues (*red*) in α1 that were replaced by their β2 counterparts. Residue numbers accord with the mature α1 and β2 subunit proteins. *E*, GABA-activated currents for α1β2γ2L heteromers and α1^A290W,(β+,int)^ homomers expressed in oocytes. GABA concentrations are indicated. *F*, GABA concentration response profiles normalized to the maximum response. EC_50_ values: α1β2γ2L, 7.1 ± 0.6 μm; α1^A290W,(β+,int)^, 18 ± 2 mm. The *inset* shows the maximum currents activated by saturating GABA. **, *p* < 0.01; *n* = 5; two-tailed unpaired *t* test.

To ascertain whether GABA activation can be imparted onto WT α1 homomers, we substituted residues at the principal (+) interface of α1 for those that are known to be important for GABA binding at the β2 subunit (+) interface (β+,int) (Fig. 7*A*) based on our structural model (Fig. 7, *B–D*). Four substitutions (S205T, E208S, V211R, and T213S = β+,int) in the α1 A290W background, α1^A290W, (β+,int)^, were selected. These new homomers were now activated by GABA but with reduced maximum currents and GABA potency compared with α1β2γ2L heteromers (Fig. 7, *E* and *F*), as might be expected by recreating the β^+^-α^−^ interface in a single α subunit.

## Discussion

The efficient delivery of neurotransmitter receptors to the cell membrane either within synaptic specialisms or to extrasynaptic locations is important for ensuring communication throughout the nervous system. Why some subunits are retained within the ER whereas others, either alone (homomers) or as heteromers, can access the cell surface is an important question. This control of expression ultimately determines the physiological and pharmacological profiles of neurotransmitter receptors. For GABA_A_Rs, we know that the ICD and ECD contain several motifs that are important for assembly and surface expression (10, 18–20). These comprise several residues located in the N terminus and are most likely expressed in the lumen of endocytic compartments and vesicles that are unlikely to be exposed to cytosolic adapter and trafficking proteins.

By comparison, our study identifies just two residues in the TMD that control the trafficking of α and γ2L subunits. Given their interfacial locations, these are likely to engage in assembly boxes between subunits. The identification of these residues allowed the pharmacological properties of α and γ2L homomers to be interrogated. A single residue exchange enabled the formation of functional α and γ2L channels without the need for auxiliary subunit co-assembly. This contrasts with WT α1 or γ2L homomers that are not expressed at the cell surface (9, 10, 36). The remarkable finding is that just single TMD residue substitutions are sufficient to bypass stringent assembly rules, preventing access to the cell surface. The importance of aromatic residues for the Ala-290 site is highlighted, as substitution of alanine with phenylalanine, tyrosine, and tryptophan all resulted in cell surface expression of α1 homomers. The size of the aromatic side chain is likely to be important, as the largest aromatic residue enabled the highest levels of subunit expression.

Our study provides a mechanism for cell surface expression of homomeric γ subunits that seemingly overrides the reported ICD ER retention sequences (10). Under physiological conditions, it is plausible that the N-terminal and ICD sequences, identified previously, operate in synchrony with the transmembrane residues identified here to ensure the assembly and targeting of heteromeric GABA_A_Rs.

Initially, we could not activate the mutant homomeric receptors with GABA_A_R ligands but could induce spontaneous activation using the 9' leucine switch for serine in the ion channel. In doing so, our results provide clear evidence that α and γ subunits expressed alone are capable of forming functional homomers on cell surface membranes following minimal molecular change. One reason why these homomers may have so far eluded detection is the lower resolution of biochemical techniques compared with electrophysiology for resolving low-affinity interactions of subunits in homomeric channel complexes.

The TMD residues that affect homomeric receptor assembly when exchanged in ρ1 subunits also revealed a significant impact on ρ1 receptor activation. The ρ1^W329A^ mutation largely removed receptor desensitization and induced faster agonist deactivation coupled to reduced GABA potency. This contrasted with ρ1^W280Q^, where GABA potency was increased along with similar desensitization but slower deactivation rates compared with WT ρ1. We created a simple but plausible receptor kinetic model that incorporated shut states with agonist (A)-unbound (R) and -bound (AR), a further shut state with agonist-bound and preactivated (AF), and finally agonist-bound open (activated) (AO) states with the facility to enter into desensitized states from the preactivated (AFD) and open (AOD) states (Model 1).

**Model 1:**
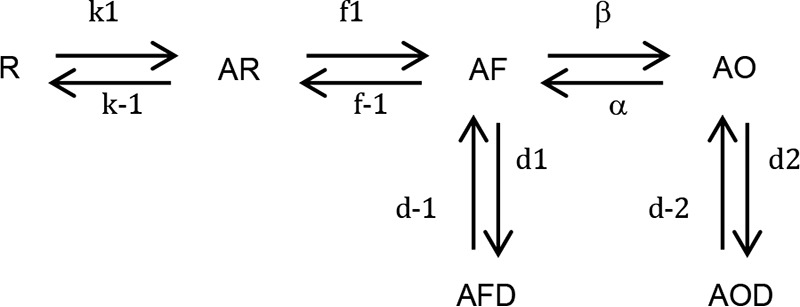


To account for the altered ρ1 receptor kinetics, the mutation W329A virtually eliminated entry into the desensitized states, AFD and AOD, and the deactivation rate (k-1) was increased by 10-fold compared with WT ρ1. For the mutant, ρ1^W280Q^, deactivation was decreased by 10-fold to account for the longer duration “tail” currents. The rate of entry into the desensitized state, AOD, was also reduced by 30%. Varying the ion channel gating constants (β and α) and the conformational constants (f1, f-1) controlling the preactivated state were unable to simply account for the altered receptor kinetics.

For the α1 subunit mutations, interestingly, Gln-241 is also a key binding residue for neurosteroids that potentiate GABA_A_R function and can, at higher concentrations, also cause direct activation (31, 35, 41). Even though spontaneous currents gated by α1^Q241W,L9'S^ remained unaffected by THDOC in HEK293 cells, in *Xenopus* oocytes, THDOC directly activated α1^A290W^ (in M3) but not α1^Q241W^ (in M1). This validates our results regarding cell surface expression and the role of Gln-241 in the neurosteroid binding site, which is present in the M3 mutant homomer but absent from the M1 mutant homomer. These results also indicate that the Gln-241 site is important for direct activation of GABA_A_Rs by THDOC. The critical role of Gln-241 for neurosteroid binding to GABA_A_Rs (35) suggested that neurosteroids could be important for trafficking by interacting with this site in the ER and Golgi compartments. Notably, only α1^Q241W^ enabled homomeric receptor expression compared with other Gln-241 substitutions. The tryptophan substitution prevents THDOC binding (35), but it also counterintuitively mimics the effect of THDOC binding by displacing the GABA concentration–response curve for αβγ GABA_A_Rs to the left (42). Nevertheless, THDOC did not initiate WT α1 subunit expression at the cell surface. Another interesting observation is that α1^Ala-290^ is also a key residue for modulation by volatile anesthetics. Its mutation prevents potentiation by enflurane (43). Thus, both α1 trafficking residues (Gln-241 and Ala-290) are important for positive allosteric modulation of GABA_A_Rs. This “dual role” is interesting because some of the intersubunit residues that are important for trafficking of heteromers are also important for ligand binding at GABA_A_Rs (44). For these interfacial residues and for at least the Gln-241 site (an endogenous ligand for Ala-290 is so far unknown), there could be an evolutionary drive to conserve these residues for heteromeric assembly.

The observation that GABA (at high concentrations) enhanced spontaneous currents in oocytes for α1 homomers and, to a lesser extent, γ2L homomers suggests that these subunits contain a rudimentary GABA-binding site that is presumably superseded by association of the β+ and α− interfaces, enabling more efficient GABA binding to αβγ heteromers. By evolving high-affinity GABA_A_Rs, rudimentary GABA binding sites on subunit homomers have presumably been superseded in heteromeric receptors. By contrast, bicuculline inhibited the spontaneous current for α1 and γ2 homomers. For zolpidem, a small block of spontaneous currents in α1^A290W^ was observed only in oocytes. It has been proposed that zolpidem, an α1 subunit-selective benzodiazepine, may bind at the α1–α1 subunit interface in αβ receptors (34), so it may be capable of modulating some α homomers.

In conclusion, N-terminal and ICD assembly sequences have been identified for α and γ subunits that enable their co-assembly into heteropentameric receptors (10, 11, 20, 24, 45). However, the new motifs identified here for of α and γ2 indicate that TMDs are equally important and rely just on single-residue exchanges for cell surface expression.

## Experimental procedures

### Cell culture and transfections

HEK-293 cells were maintained at 37 °C in 95% air and 5% CO_2_ in Dulbecco's modified Eagle's medium supplemented with 10% v/v fetal calf serum, 100 units/ml penicillin-G, 100 μg/ml streptomycin, and 2 mm l-glutamine. All medium components were obtained from Life Technologies unless otherwise stated. Cells were plated onto 22-mm glass coverslips (VWR), coated with poly-l-lysine (Sigma), and transfected with cDNAs encoding for GABA_A_R subunits along with eGFP using a calcium phosphate method applied 2–3 h after plating (25).

### cDNAs and constructs

pEGFP-C1, murine WT myc-tagged α1, β2, γ2L, and human ρ1 GABA_A_R cDNAs subcloned into pRK-5 have all been described previously (1, 2 and were used for the chimeric α1–ρ1 constructs (46) (α1–ρ1^M1–4^, α1–ρ1^M1/2^, α1–ρ1^M3–4^, α1–ρ1^ICD2-M4^, α1^BBS^–ρ1^M4^, α1–ρ1^M1/M3^, α1–ρ1^M1^, and α1–ρ1^M3^). A mimotope of 13 amino acids encoding the BBS was inserted between amino acids 4 and 5 of the mature α1 subunit protein using an inverse PCR adjacent to a myc tag (Table S1). Gln-241 and Ala-290 of the mature BBS-tagged α1 protein were mutated to single amino acid mutants. Single M1 and M3 residues in α1 were mutated to C233A (α1^C233A^), M235L (α1^M235L^), C292S (α1^C292S^), and a triple mutant containing S298L, A299S, and L300V (α1^SAL-LSV^). For α1 subunits, intracellular domain 1 (ICD1, between M1–M2, including amino acids Asn-247–Glu-249) and intracellular domain 2 (ICD2, between M3–M4, including amino acids Thr-310–Lys-390) were serially replaced with the GLIC intracellular loop sequence -SQPARAA- using a similar inverse PCR-based mutagenesis approach to provide α1-ICD1^GLIC^ and α1-ICD2^GLIC^. A double ICD1–2^GLIC^, where both ICDs were replaced with the GLIC heptapeptide, was created using α1-ICD2^GLIC^ as template and primers for α1-ICD1^GLIC^. Similar strategies were employed to replace ICD1 (between Asn-247–Ser-250) and/or ICD2 with a multiple glycine-serine (-GGSSGGSS-) flexible linker. The 9'L in the M2 lining of α1 was substituted for a serine (L9'S) using a Kunkel PCR strategy (47). Four amino acids (S205T, E208S, V211R, and T213S) in α1^A290W^ at the principle (+) interface were replaced by corresponding residues from the β (+) interface to recreate the GABA-binding interface (α1^A290W,(β+, int)^). Ser-301 in γ2L M3 was mutated to tryptophan (γ2L^S301W^), and the 9' serine in γ2L M2 was substituted for a leucine residue (γ2L^L9'S^) using an inverse PCR approach. Trp-280 in M1 and Trp-329 in M3 of ρ1 were substituted for glutamine (ρ1^W280Q^) and alanine (ρ1^W329A^), respectively. BBS tags were inserted into the α1-ρ1 chimeras (α1^BBS^-ρ1^M1–4^, α1^BBS^-ρ1^M1/2^, α1^BBS^-ρ1^M3–4^, α1^BBS^-ρ1^ICD2-M4^, α1^BBS^-ρ1^M4^, α1^BBS^-ρ1^M1/M3^, α1^BBS^-ρ1^M1^, and α1^BBS^-ρ1^M3^) using restriction digestion, with SpeI and PpuMI ligating the BBS tag containing the α1^BBS^ N terminus fragment with the respective chimeras.

### Whole-cell patch clamp electrophysiology

Whole-cell currents were recorded from transfected HEK293 cells 48 h after transfection by voltage clamp at −30 mV with optimized series resistance (Rs < 10 megaohms) and whole-cell membrane capacitance compensation. Borosilicate glass patch electrodes (resistance of 4–5 megaohms) were filled with an internal solution containing 120 mm CsCl, 1 mm MgCl_2_, 11 mm EGTA, 30 mm KOH, 10 mm HEPES, 1 mm CaCl_2_, and 2 mm K_2_ATP (pH 7.2). HEK293 cells were superfused with a saline solution containing 140 mm NaCl, 4.7 mm KCl, 1.2 mm MgCl_2_, 2.52 mm CaCl_2_, 11 mm glucose, and 5 mm HEPES (pH 7.4). Membrane currents were filtered at 5 kHz (−3 db, 6th pole Bessel, 36 db per octave).

Picrotoxin inhibition relationships for the spontaneous membrane currents were generated by measuring the current (I) at each picrotoxin concentration and normalizing these to the maximum inhibition achieved by 1 mm picrotoxin (I_max_) before fitting the concentration response relationship with,

(Eq. 1) $$\text{I}/{\text{I}}_{\text{max}}=1-\left[\left(1/1+{\left({\text{IC}}_{50}/\left[\text{B}\right]\right)}^{\text{n}}\right)\right]$$

where B is the concentration of picrotoxin, IC_50_ is the concentration of picrotoxin causing 50% maximum inhibition of the spontaneous current, and n is a slope factor.

Concentration response relationships for GABA-activated membrane currents were generated by measuring the current (I) at each GABA concentration and normalizing these currents to the maximal GABA current (I_max_). The concentration response relationship was fitted with the Hill equation,

(Eq. 2) $$\text{I}/{\text{I}}_{\text{max}}=\left(1/{\left(1+{\text{EC}}_{50}/\left[\text{A}\right]\right)}^{\text{n}}\right)$$

where A is the concentration of GABA, EC_50_ is the concentration of GABA causing 50% of the maximal GABA current, and n is the slope. All concentration response data were curve fitted in Origin (version 6).

The extent of desensitization was calculated for ρ1 receptors at 60 s and for αβγ heteromers at 20 s following application of 1 mm (saturating) GABA. The residual current was normalized to the peak GABA current measured immediately after the start of GABA application, which was defined as 0% (*i.e.* no desensitization).

The deactivation of ρ1 receptors was calculated by applying 1 mm GABA to activate the receptors and allowing desensitization to reach steady state at ∼60 s, at which point GABA application was stopped. The deactivating currents (I) were normalized to the time point at which GABA application was stopped (I_max_), and the curves were fitted to a bi-exponential function,

(Eq. 3) $$\text{I}/{\text{I}}_{\text{max}}=-100+{\text{A}}_{1}\left(1-{\text{e}}^{-\text{t}/\tau 1}\right)+{\text{A}}_{2}\left(1-{\text{e}}^{-\text{t}/\tau 2}\right)$$

where t is time, A_1_ and A_2_ are areas of the individual exponential functions, and τ1 and τ2 are the exponential time constants. A weighted time constant was reported.

### Two-electrode voltage clamp

All procedures on animals were carried out in accordance with the Animals (Scientific Procedures) Act 1986 and European Union directives on the use of animals for scientific research. *Xenopus laevis* oocytes were obtained by removing ovaries from frogs, followed by incubation for 2–3 h in collagenase type I (Worthington) in OR2 solution containing 85 mm NaCl, 5 mm HEPES, and 1 mm MgCl_2_ (pH 7.6, adjusted with KOH). Defolliculated oocytes were washed in OR2 and maintained at 18 °C in Barth's solution containing 88 mm NaCl, 1 mm KCl, 0.33 mm Ca(NO_3_)_2_, 0.41 mm CaCl_2_, 0.82 mm MgSO_4_, 2.4 mm NaHCO_3_, and 10 mm HEPES (pH adjusted to 7.6 with NaOH). Oocytes were injected with 27.6 nl of GABA_A_R homomers at concentrations of 30–60 ng/μl and used for recordings 2–5 days after injection.

Two-electrode voltage clamp recordings were performed at room temperature by superfusing oocytes in a recording solution containing 100 mm NaCl, 2 mm KCl, 2 mm CaCl_2_, 1 mm MgCl_2_, and 5 mm HEPES (pH adjusted to 7.4 with NaOH) using an Axoclamp 2B amplifier, a Digidata 1322A interface, and pClamp 8 (Molecular Devices). Oocytes were voltage-clamped at −60 to −110 mV, and currents were digitized at 500 Hz and filtered at 50 Hz.

### Fluorescent α-BgTx staining

HEK293 cells were studied 48 h after transfection and washed with Krebs solution containing 140 mm NaCl, 4.7 mm KCl, 1.2 mm MgCl_2_, 2.52 mm CaCl_2_, 11 mm glucose, and 5 mm HEPES (pH 7.4) and incubated in 400 nm α-BgTx coupled with Alexa Fluor 555 (α-BgTx–AF555; Life Technologies) for 10 min at room temperature, followed by washing and then fixation in 4% v/v paraformaldehyde (Sigma) for 10 min at room temperature. The cells were imaged immediately post-fixation in saline using a Zeiss LSM 510 Meta confocal microscope and an Achroplan ×40 water differential interference contrast objective (numerical aperture (NA) 0.8) as described previously (48). This involved choosing the optimal z-section and acquiring individual images as a mean from 4 scans in 16 bits using a 543-nm helium–neon laser and a 560-nm long-pass filter for α-BgTx–AF555 and a 488 argon laser with a 505- to 530-nm band pass filter for eGFP. In experiments requiring membrane permeabilization, cells were labeled with α-BgTx–Alexa Fluor 488, followed by fixation with 4% v/v paraformaldehyde for 10 min at room temperature before serial washing (three times) in PBS (Sigma) and application of 0.1% w/v Triton X-100 (Sigma) for 10 min at room temperature in 10% v/v fetal calf serum. Cells were washed to remove the detergent, and 400 nm α-BgTx–AF555 was applied for 30 min at room temperature to label intracellular receptors along with a rabbit primary antibody against the endoplasmic reticulum marker calnexin (Abcam, ab22595). Cells were washed and incubated in a goat anti-rabbit Cy5 antibody (Life Technologies, A10523) before mounting in ProlongGold (Life Technologies) and used for imaging.

### Immunolabeling of HEK293 cells

48 h after transfection, cells were washed with Krebs solution at 4 °C and incubated in primary antibodies against γ2 (Synaptic Systems, 224 004) or ρ1 (Abcam, ab85667) for 30 min at 4 °C, followed by washes, fixation, and incubation in secondary antibodies (goat anti-guinea pig or rabbit Alexa Fluor 555) at room temperature for 30 min. Cells were washed and imaged immediately after labeling with secondary antibodies.

### Image analysis

Confocal images were analyzed using ImageJ (version 1.410) as described previously (25). For each cell, the cell surface (CS) membrane was identified by defining a region of interest (ROI) in the eGFP channel; this was transferred to the α-BgTx–AF555 channel (ROI_CS_), and mean membrane fluorescence values were determined. Mean background (b) fluorescence was determined from another ROI selected so that it was devoid of cells (ROI_b_). This was subtracted from the mean membrane fluorescence for the cell surface (ROI_CS_), providing a mean corrected fluorescence intensity value (ΔROI = ROI_CS_ − ROI_b_). These ΔROI values, for different combinations of receptors, were graphically plotted using Origin.

### Homology modeling

The primary sequences for murine α1, β2, and γ2L subunits were aligned using ClustalW (49). The mature heteropentameric GABA_A_R was subsequently modeled based on the crystal structure template for the GABA β3 subunit homomer (PDB code 4COF) using Modeler 9 version 7 (50). The models with the lowest discrete optimized protein energy scores were used, and optimal side-chain configurations were determined with SCWRL4 (51). All structural images were visualized and rendered using the PyMOL molecular graphics system (DeLano, LLC) (40).

## Author contributions

T. G. S. and S. H. conceptualization; T. G. S. and S. H. formal analysis; T. G. S. funding acquisition; T. G. S. and S. H. writing-original draft; T. G. S. and S. H. project administration; T. G. S. and S. H. writing-review and editing; S. H. investigation.

## Supplementary Material

Supporting Information

## Footnotes

3 The abbreviations used are:

GABA_A_R: type A GABA receptor
HEK: human embryonic kidney
ECD: extracellular domain
BgTx: α-bungarotoxin
BBS: BgTx-binding site
TMD: transmembrane domain
ICD: intracellular domain
AF555: Alexa Fluor 555
ER: endoplasmic reticulum
THDOC: tetrahydrodeoxycorticosterone
cDNA: complementary DNA
eGFP: enhanced GFP
CS: cell surface
ROI: region of interest
ANOVA: analysis of variance
PTX: picrotoxin
PB: pentobarbitone
PS: pregnenolone sulfate
Bic: bicuculline.
