# Supplementary material for: Cell surface expression of homomeric GABAA receptors depends on single residues in subunit transmembrane domains
Source: J Biol Chem. 2018 Jul 9;293(35):13427–39. doi: 10.1074/jbc.RA118.002792 (PMC6120189; doi:10.1074/jbc.RA118.002792)
Supplement: Supporting Information [file supp_293_35_13427__index.html]

Cell surface expression of homomeric GABAA receptors depends on single residues in subunit transmembrane domains — GABAA receptor trafficking — Cell surface expression of homomeric GABAA receptors depends on single residues in subunit transmembrane domains — GABAA receptor trafficking — Cell surface expression of homomeric GABAA receptors depends on single residues in subunit transmembrane domains — GABAA receptor trafficking — Supporting Information 

# Cell surface expression of homomeric GABAA receptors depends on single residues in subunit transmembrane domains

## Supporting Information

- Supplementary Information - Supplementary Figures and Table
