# Supplementary material for: Cell surface expression of homomeric GABAA receptors depends on single residues in subunit transmembrane domains
Source: J Biol Chem. 2018 Jul 9;293(35):13427–39. doi: 10.1074/jbc.RA118.002792 (PMC6120189; doi:10.1074/jbc.RA118.002792)
Supplement: Supporting Information [file supp_RA118.002792_136520_2_supp_162440_pb8xk0.pdf]

Supporting Information

Single transmembrane domain residues control cell surface expression of homomeric $GABA_A$ receptors

Saad Hannan and Trevor G. Smart

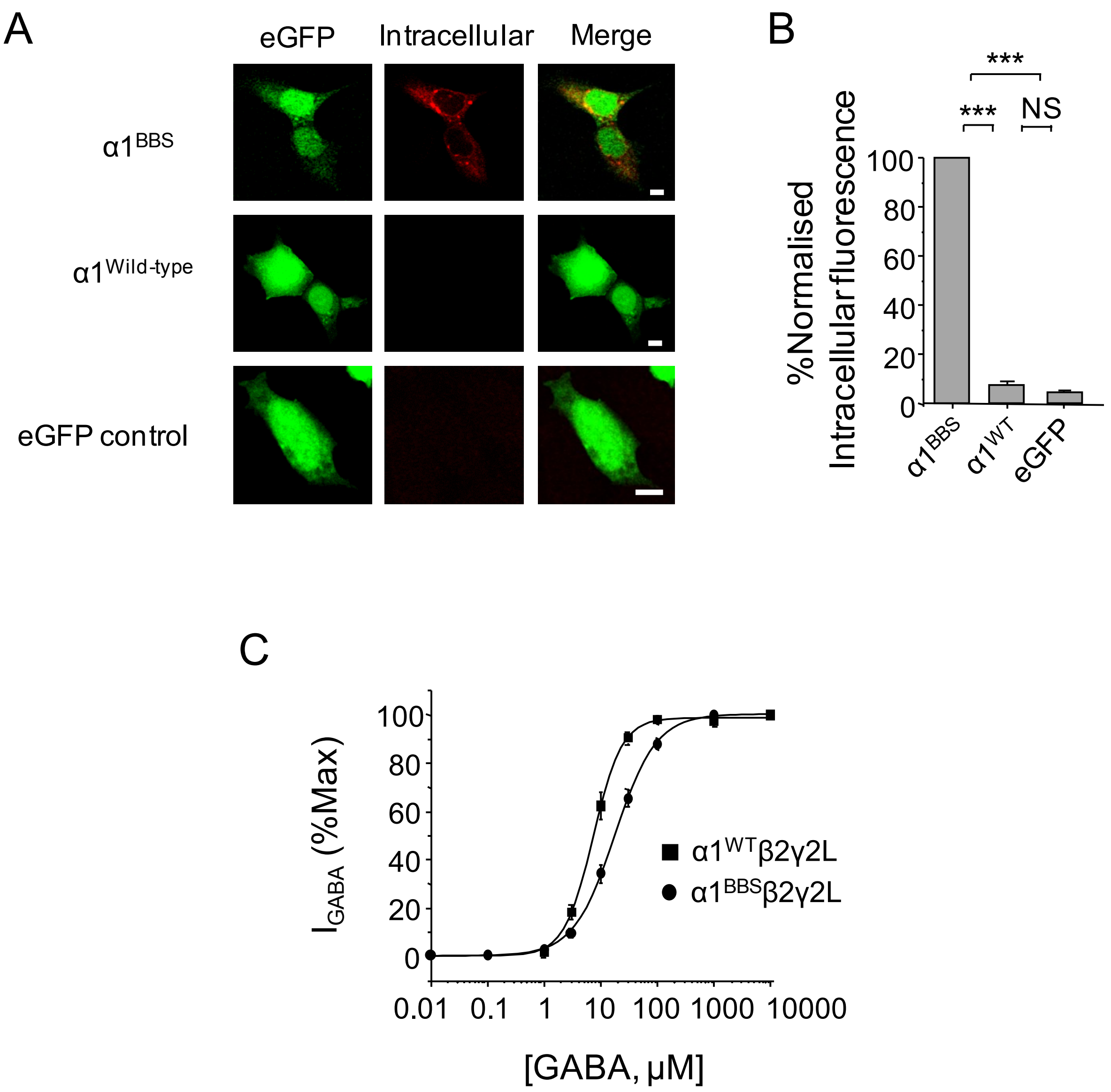

Fig. S1. Inserting the α-bungarotoxin binding site allows specific labeling of α1 subunits

(A), Confocal images show intracellular labelling in permeabilised HEK293 cells expressing α-bungarotoxin (α-BgTx) binding site (BBS) containing α1 subunits, wild-type α1 subunits or eGFP controls. Note that labelling by α-BgTx Alexa Fluor 555 is only present when the BBS-containing receptors are expressed. (B), Intracellular fluorescence for wild-type and BBS-tagged α1 subunits normalised to $\alpha 1^{BBS}$. (C), GABA concentration response curves for wild-type and BBS-tagged α1 subunit containing $GABA_A$ receptors. ***P<0.001, NS – not significant. n = 5 - 12, one-way ANOVA. Scale bars = 5 µm. In this and all proceeding figures, bars represent mean ± s.e.m.

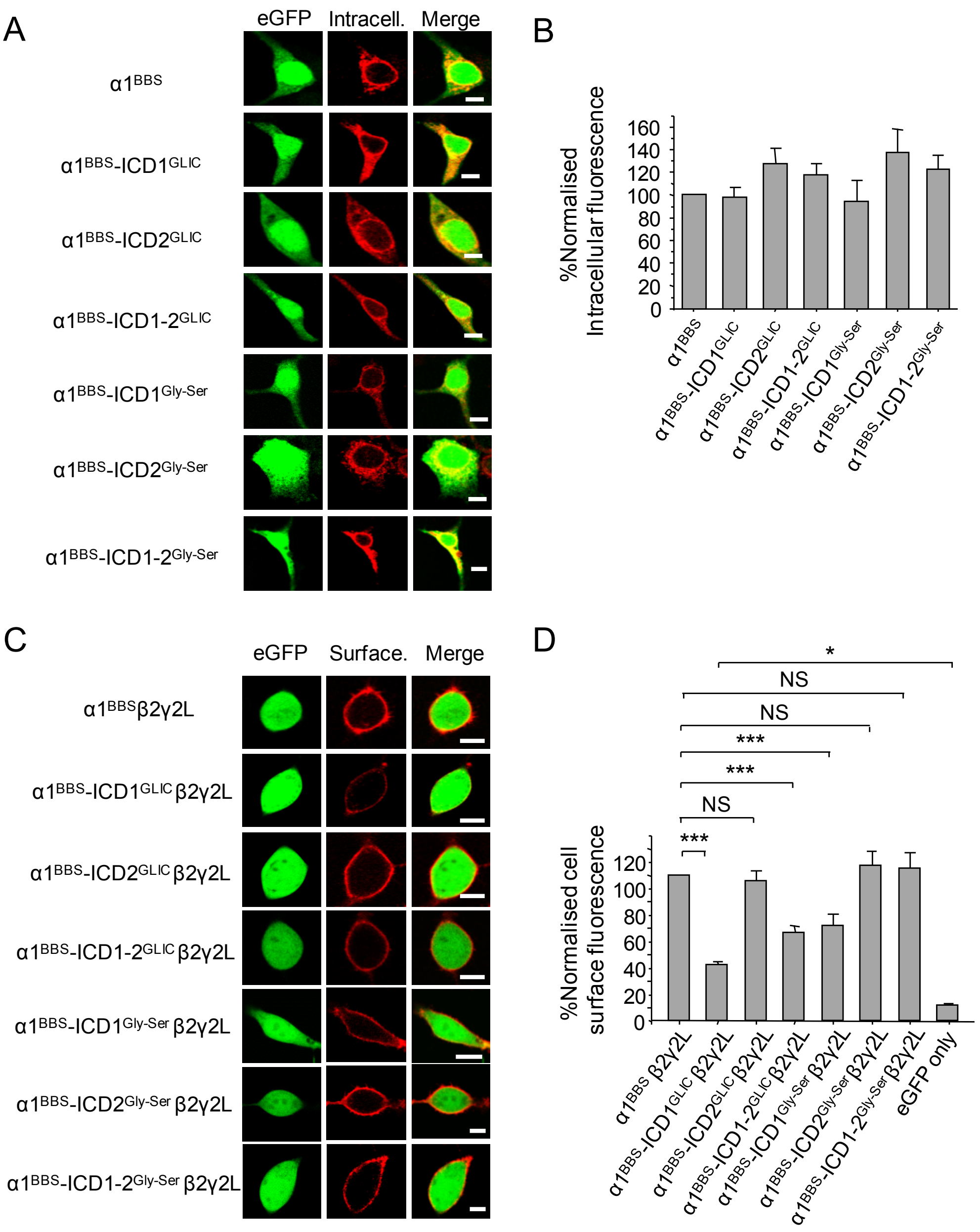

Fig. S2. Expression of α1^{BBS}-ICD^{GLIC} and α1^{BBS}-ICD$^{Gly-Ser}$ chimeras

(A), Confocal images showing intracellular labelling of α1^{BBS} and chimeras of α1^{BBS} containing a GLIC sequence (-SQPARAA-) or a Glycine-Serine (Gly-Ser) flexible linker replacing the first intracellular domain (ICD) 1 between M1-2 (α1^{BBS}-ICD1^{GLIC}; α1^{BBS}-ICD1$^{Gly-Ser}$) or second ICD2 between M3-4, or both ICD1 and ICD2, in permeabilised HEK293 cells. (B) Bar chart of intracellular labelling for α1^{BBS}-GLIC and α1^{BBS}-Gly-Ser chimeras. Data have been normalised to the intracellular fluorescence levels for α1^{BBS} subunits. (C) Confocal images of cell surface labelling for α1^{BBS}-GLIC and α1^{BBS}-Gly-Ser chimeras expressed with β2γ2L. (D), Bar chart of cell surface labelling for chimeric α1^{BBS}-GLIC and α1^{BBS}-Gly-Ser heteromers with βγ subunits. Data have been normalised to the cell surface fluorescence levels for α1^{BBS}β2γ2L receptors.*P<0.05, ***P<0.001, NS – not significant, n = 14-32, one-way ANOVA. Scale bars = 5 µm.

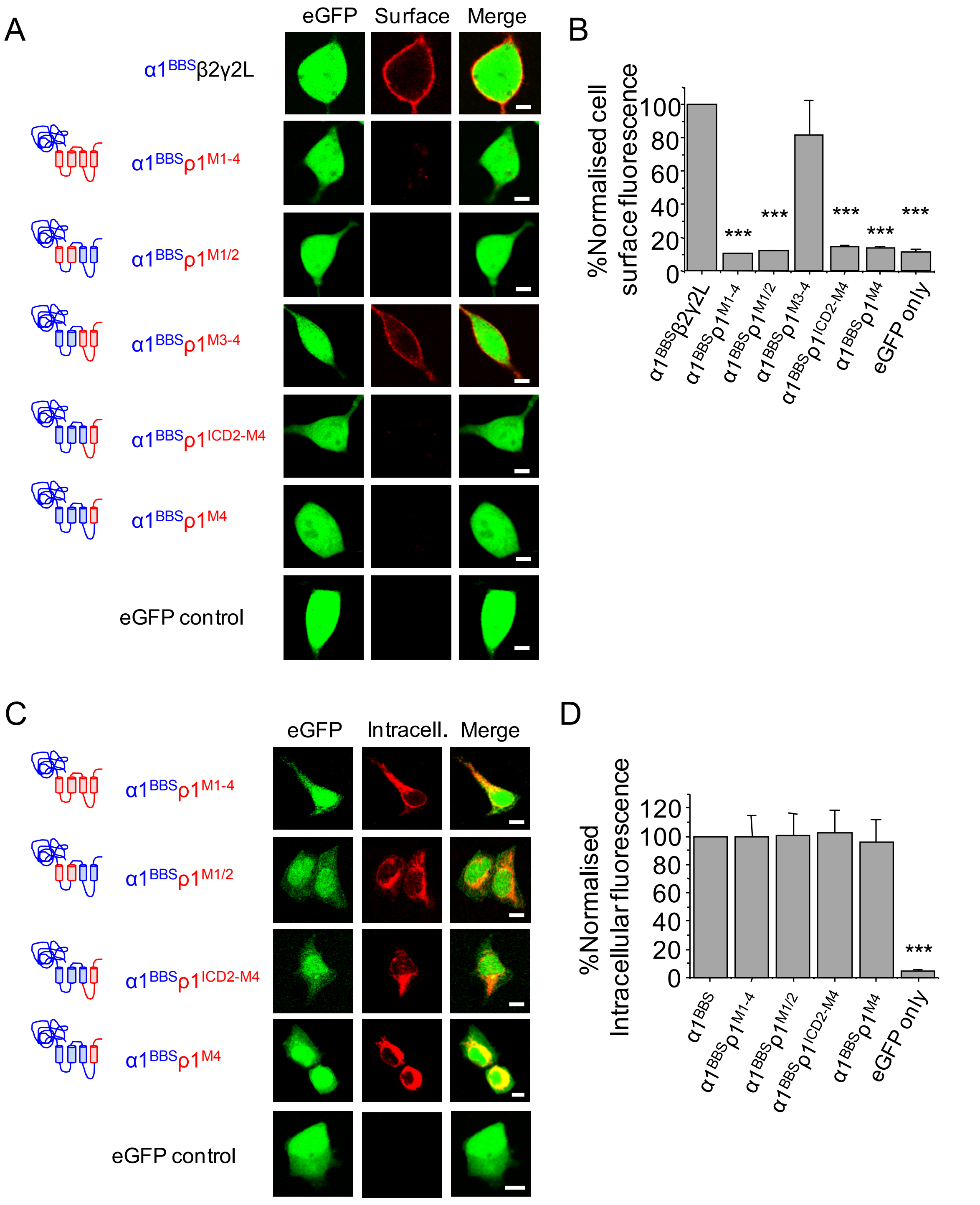

Fig. S3. Expression of α1-ρ1 chimeras

(A), Confocal images of cell surface labelling for $\alpha1^{BBS}$-ρ1 chimeras in HEK293 cells. (B), Bar chart of cell surface labelling for $\alpha1^{BBS}$-ρ1 chimeras. Data have been normalised to the cell surface fluorescence levels for $\alpha1^{BBS}\beta2\gamma2L$ receptors. (C), Confocal images showing intracellular labelling of α1-ρ1 chimeras in permeabilised HEK293 cells. (D), Bar chart of intracellular labelling of α1-ρ1 chimeras. Data are normalised to the intracellular fluorescence levels of $\alpha1^{BBS}$ receptors. ***$P<0.001$, n = 5 - 13, one-way ANOVA. Scale bars = 5 µm.

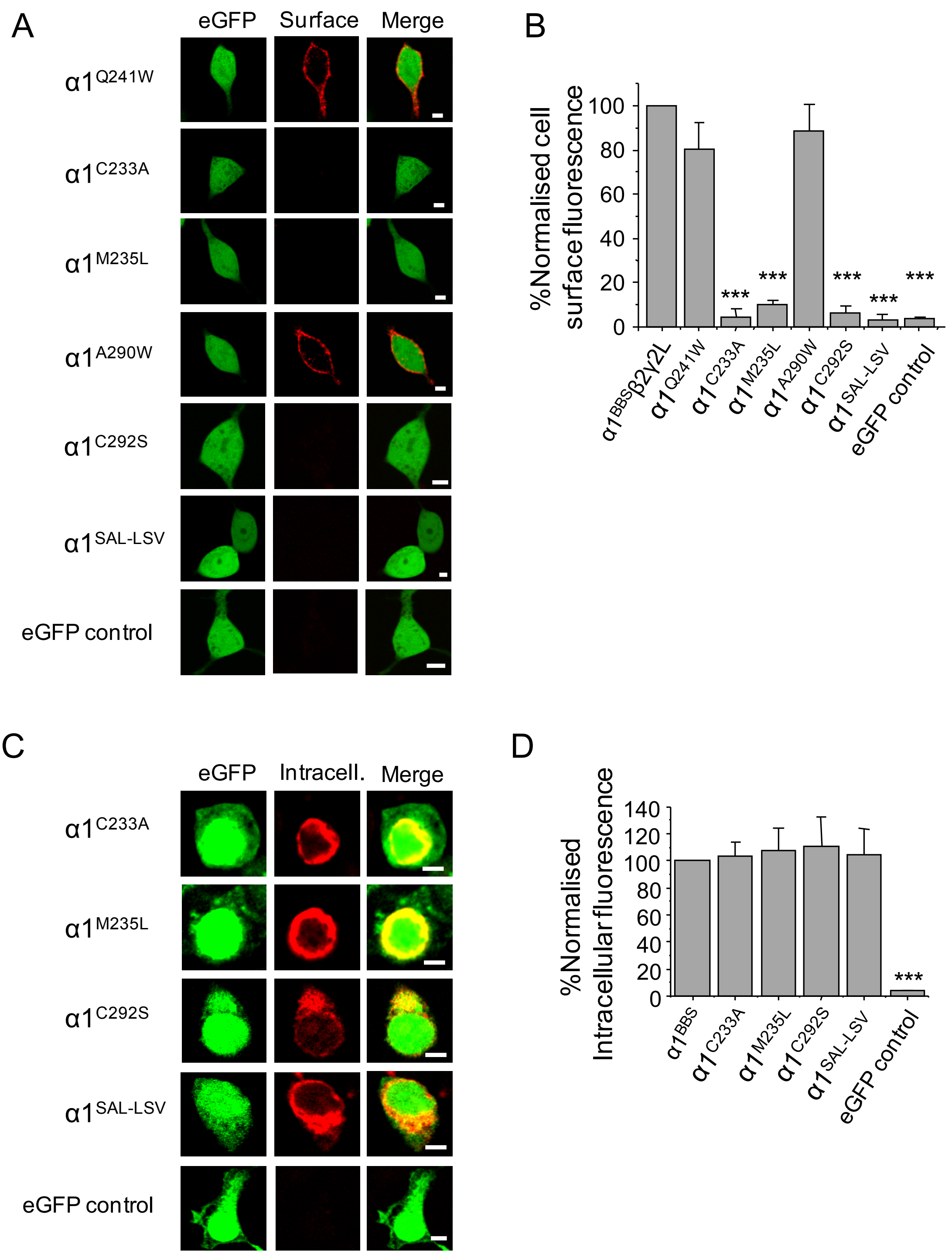

Fig. S4. Expression of α1 M1 and M3 mutants

(A), Confocal images showing cell surface labelling of α1 M1 or M3 mutants expressed in HEK293 cells. (B), Bar chart of cell surface labelling of α1 M1 or M3 mutants. Data are normalised to fluorescence levels for $\alpha1^{BBS}\beta2\gamma2L$ receptors. (C), Confocal images showing intracellular labelling of α1 M1 or M3 residue mutants in permeabilised HEK293 cells. (D), Bar chart of intracellular labelling for α1 M1 or M3 mutants. Data are normalised to intracellular fluorescence levels of $\alpha1^{BBS}$ receptors. ***$P<0.001$, $n = 6 - 11$, one-way ANOVA. Scale bars = 5 μm.

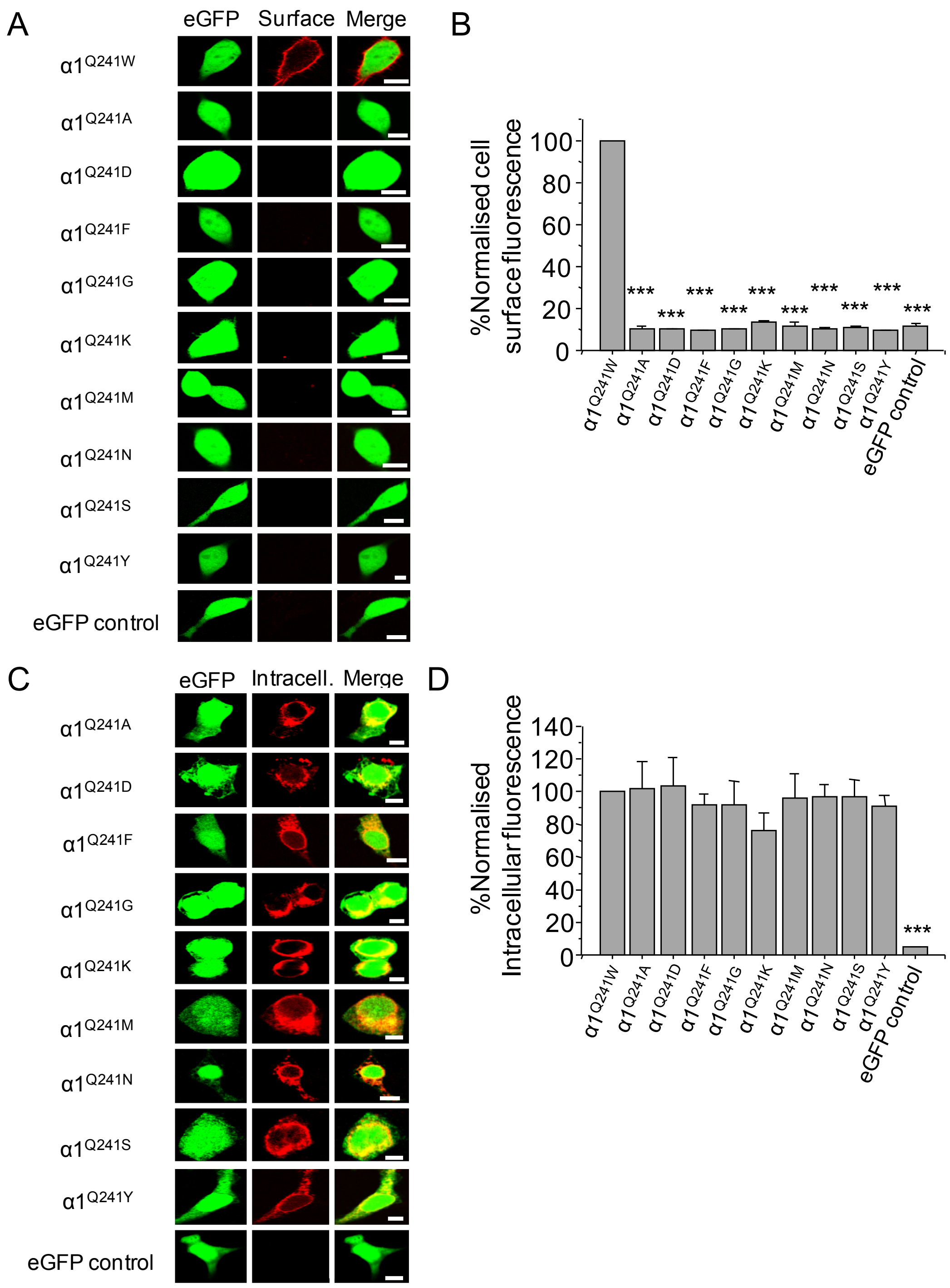

Fig. S5. Expression of α1 Q241 mutants

(A), Confocal images showing cell surface labelling of multiple α1 Q241 mutants expressed in HEK293 cells. (B), Bar chart of cell surface labelling of α1 Q241 mutants. Data are normalised to cell surface fluorescence levels of $\alpha1^{BBS,Q241W}$ receptors. (C), Confocal images showing intracellular labelling of α1 Q241 mutants in permeabilised HEK293 cells. (D), Bar chart of intracellular labelling of α1 Q241 mutants. Data are normalised to intracellular fluorescence levels of $\alpha1^{BBS,Q241W}$. ***$P<0.001$, n = 7 - 29, one-way ANOVA. Scale bars = 5 μm.

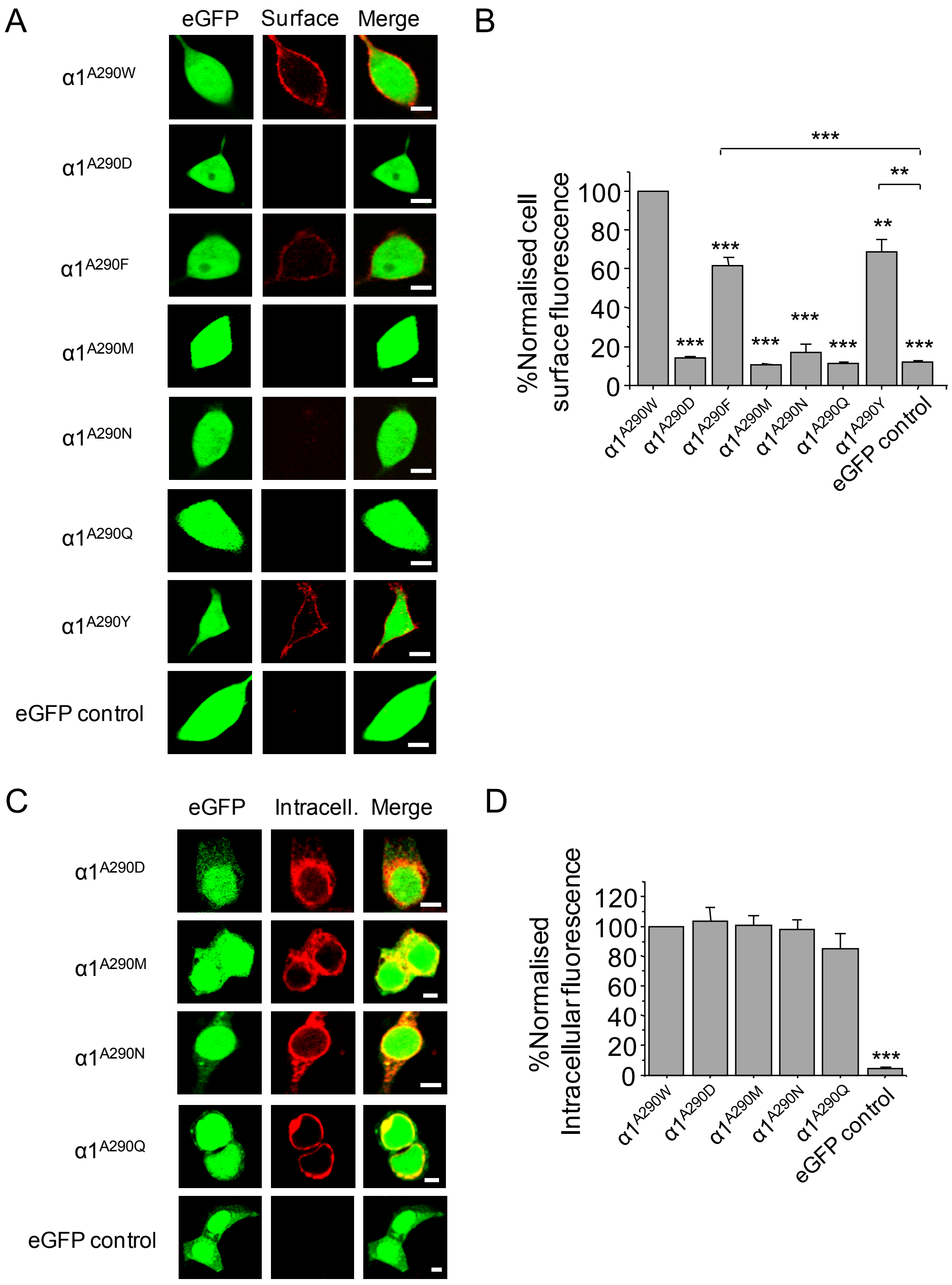

Fig. S6. Expression of α1 A290 mutants

(A), Confocal images showing cell surface labelling of multiple α1 A290 mutants in HEK293 cells. (B), Bar chart of cell surface labelling of α1 A290 mutants. Data are normalised to cell surface fluorescence levels of $\alpha1^{BBS,A290W}$ receptors. (C), Confocal images showing intracellular labelling of α1 A290 mutants in permeabilised HEK293 cells. (D), Bar chart of intracellular labelling of α1 A290 mutants. Data are normalised to intracellular fluorescence levels of $\alpha1^{BBS,A290W}$. ***P<0.001, n = 6 - 12, one-way ANOVA. Scale bars = 5 μm.

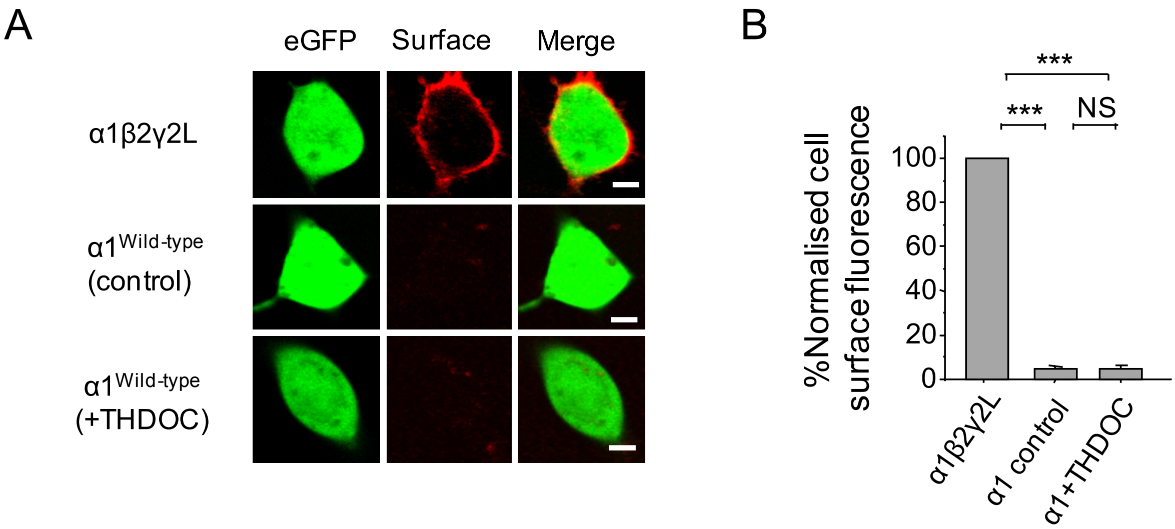

Fig. S7. THDOC does not enable cell surface expression of α1 subunits

(A), Confocal images showing cell surface labeling of $\alpha1^{BBS}\beta2\gamma2L$ or $\alpha1^{BBS}$ homomers in the presence or absence of 10 μM THDOC in HEK293 cells. Cells were incubated with THDOC for 36 - 48 hr. (B), Cell surface fluorescence for $\alpha1^{BBS}\beta2\gamma2L$ and $\alpha1^{BBS}$ in control or in THDOC. ***P<0.001, NS – not significant. n = 5, one-way ANOVA. Scale bars = 5 μm.

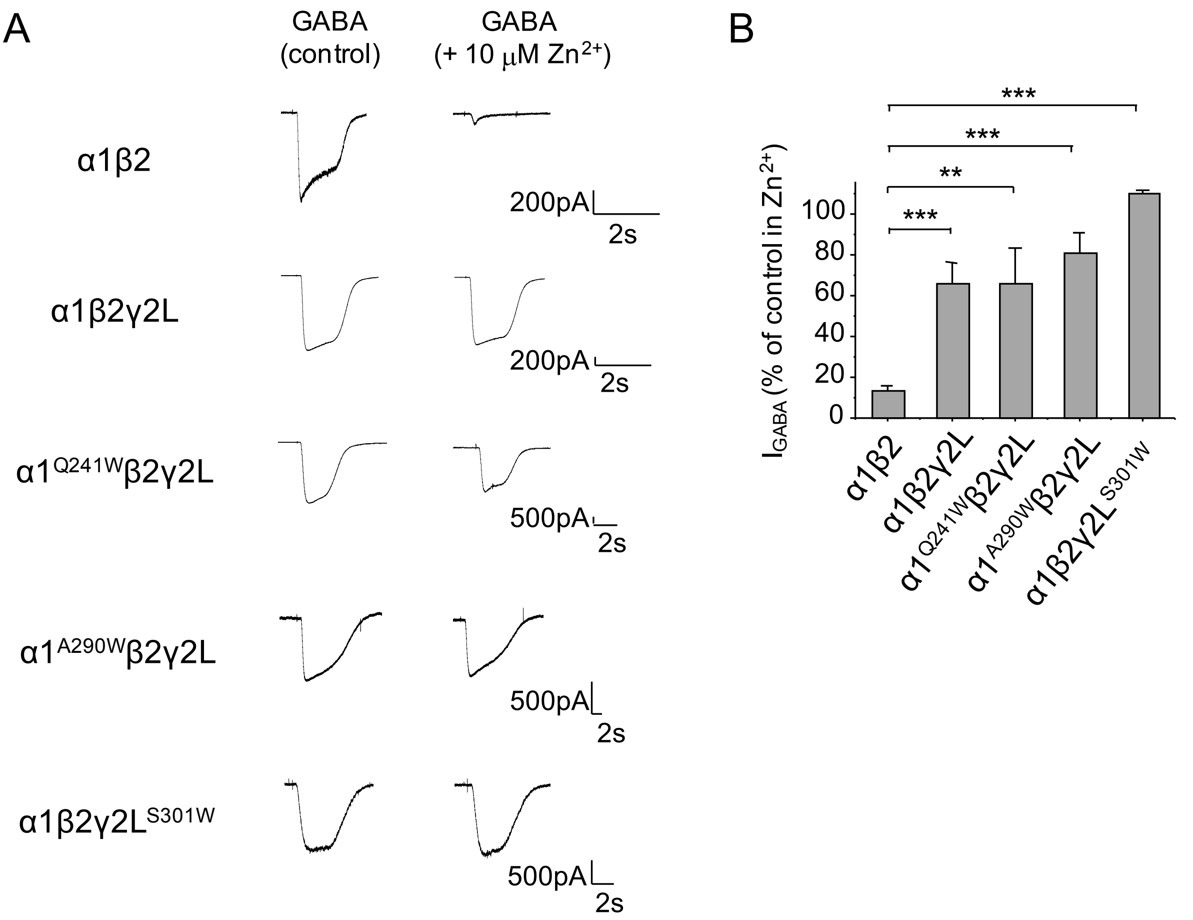

Fig. S8. Zn^{2+} sensitivity of GABA-activated currents for αβγ heteromers

(A) Representative GABA-activated currents recorded from wild-type α1β2 and α1β2γ2L heteromers, and from mutant α1β2γ2L receptors expressed in HEK293 cells in the presence or absence of 10 μM Zn^{2+}. (B), Bar chart showing GABA-activated current (%) that remains in the presence of Zn^{2+} compared to control. ***P<0.001, **P<0.01, n = 3 - 8, one-way ANOVA.

Table S1 – Primer sequences (5' – 3') used for mutagenesis

Construct	Forward Primer	Reverse Primer	Strategy
$\alpha1^{BBS}$	GTTTAGAACCATATCCAGATGATGAAC TTAAAGACAACACCA	TACTTTCATAATATCTCCATAGGTCT TCTTCTGATATTAG	Inverse
$\alpha1^{Q241W}$	TGGGTCTCCTTCTGGCTCAACAGAGAG T	GGAGAGAATAACTGTCATTATGCAC G	Inverse
$\alpha1^{Q241A}$	GCAGTCTCCTTCTGGCTCAACAGAGAG T	GGAGAGAATAACTGTCATTATGCAC G	Inverse
$\alpha1^{Q241D}$	GACGTCTCCTTCTGGCTCAACAGAGAG T	GGAGAGAATAACTGTCATTATGCAC G	Inverse
$\alpha1^{Q241F}$	TTCGTCTCCTTCTGGCTCAACAGAGAG T	GGAGAGAATAACTGTCATTATGCAC G	Inverse
$\alpha1^{Q241G}$	GGAGTCTCCTTCTGGCTCAACAGAGAG T	GGAGAGAATAACTGTCATTATGCAC G	Inverse
$\alpha1^{Q241K}$	AAGGTCTCCTTCTGGCTCAACAGAGAG T	GGAGAGAATAACTGTCATTATGCAC G	Inverse
$\alpha1^{Q241M}$	ATGGTCTCCTTCTGGCTCAACAGAGAG T	GGAGAGAATAACTGTCATTATGCAC G	Inverse
$\alpha1^{Q241N}$	AACGTCTCCTTCTGGCTCAACAGAGAG T	GGAGAGAATAACTGTCATTATGCAC G	Inverse
$\alpha1^{Q241S}$	TCAGTCTCCTTCTGGCTCAACAGAGAG T	GGAGAGAATAACTGTCATTATGCAC G	Inverse
$\alpha1^{Q241Y}$	TACGTCTCCTTCTGGCTCAACAGAGAG T	GGAGAGAATAACTGTCATTATGCAC G	Inverse
$\alpha1^{A290W}$	TGGGTATGCTATGCCTTTGTTTTCTC	AATAAACCAGTCCATAGCTGTTGC	Inverse
$\alpha1^{A290D}$	GACGTATGCTATGCCTTTGTTTTCTC	AATAAACCAGTCCATAGCTGTTGC	Inverse
$\alpha1^{A290F}$	TTCGTATGCTATGCCTTTGTTTTCTC	AATAAACCAGTCCATAGCTGTTGC	Inverse
$\alpha1^{A290M}$	ATGGTATGCTATGCCTTTGTTTTCTC	AATAAACCAGTCCATAGCTGTTGC	Inverse
$\alpha1^{A290N}$	AACGTATGCTATGCCTTTGTTTTCTC	AATAAACCAGTCCATAGCTGTTGC	Inverse
$\alpha1^{A290Q}$	CAGGTATGCTATGCCTTTGTTTTCTC	AATAAACCAGTCCATAGCTGTTGC	Inverse
$\alpha1^{A290Y}$	TACGTATGCTATGCCTTTGTTTTCTC	AATAAACCAGTCCATAGCTGTTGC	Inverse
$\alpha1^{C233A}$	GCCATAATGACAGTTATTCTCTCCC	CGGCAGATATGTTTGAATAACAAAG	Inverse
$\alpha1^{M235L}$	CTGACAGTTATTCTCTCCCAAGTC	TATGCACGGCAGATATGTTTGAATA A	Inverse
$\alpha1^{C292S}$	CCTATGCCTTTGTTTTCTCAGCTCTG	ATACTGCAATAAACCAGTCCATAGC	Inverse
$\alpha1^{SAL\text{-}LSV}$	TATCTGTGATTGAGTTTGCCACAGTAA ACTA	AGAAAACAAAGGCATAGCATACTGC	Inverse
$\alpha1^{L9'S}$	GTCTTTGGAGTGACGACTGTTTCGACT ATGACAACCTTGAGTATCAG	CTGATACTCAAGGTTGTCATAGTCGA AACAGTCGTCACTCCAAAGAC	Kunkel
α1-ICD1^{GLIC}	CAAGAGCAGCATCAGTACCAGCAAGA ACTGTCTTTGGAGTG	CTGGTTGTGAGAGCCAGAAGGAGAC TTGGGAGAGAATAACT	Inverse
α1-ICD2^{GLIC}	AAGAGCAGCACTGTCAAGAATAGCCT TTCCGCTG	GCTGGTTGTGATACTGTGGCAAACTC AATCA	Inverse
α1-ICD1$^{Gly\text{-}Ser}$	GAGGATCATCAGTACCAGCAAGAACT GTCTTTGGAGTGA	CTGATGATCCTCCGAGCCAGAAGGA GACTTGGGAGAGAAT	Inverse
α1-ICD2$^{Gly\text{-}Ser}$	GAGGATCATCAATCGACCGACTGTCA AGAATAGCCTTTC	CTGATGATCCTCCGAAATAGTTTACT GTGGCAAACTCAATCA	Inverse
$\alpha1^{A290W,(\beta+, int)}$	GTGCGTATGAGCACTCACTTCCACTTG AAGAGAAAAATTGG	ATATGATCCAGTAGTGGACTGAACA ATTCCAGAGTCAAC	Inverse
$\gamma2L^{S301W}$	TGGGTATGCTATGCCTTTGTTTTCTC	AATAAACCAGTCCATAGCTGTTGC	Inverse
$\gamma2L^{L9'S}$	TCGACCATGACAACTTTAAGCACC	GACAGTAGTGATTCCTAAAGATGTT	Inverse
$\rho1^{W280Q}$	CAGGTGTCCTTCTGGATCGACCGC	GGACAGCATGACCATCAGGGTAG	Inverse
$\rho1^{W329A}$	GCGGTCAGCTTTGTGTTCGTGTTCC	GAGGTAGATGTCCACGGCCTTG	Inverse
